# Resistance exercise stimulates mixed muscle protein synthesis in lean and obese young adults

**DOI:** 10.14814/phy2.13799

**Published:** 2018-07-16

**Authors:** Carl J. Hulston, Rachel M. Woods, Rebecca Dewhurst‐Trigg, Sion A. Parry, Stephanie Gagnon, Luke Baker, Lewis J. James, Oonagh Markey, Neil R. W. Martin, Richard A. Ferguson, Gerrit van Hall

**Affiliations:** ^1^ School of Sport, Exercise & Health Sciences Loughborough University Loughborough Leicestershire United Kingdom; ^2^ Clinical Metabolomics Core Facility Department of Clinical Biochemistry Rigshospitalet Department of Biomedical Sciences University of Copenhagen Copenhagen Denmark; ^3^ Present address: Sion A. Parry Oxford Centre for Diabetes, Endocrinology and Metabolism University of Oxford Churchill Hospital Oxford United Kingdom

**Keywords:** Anabolic resistance, exercise, obesity

## Abstract

Obese individuals exhibit a diminished muscle protein synthesis response to nutrient stimulation when compared with their lean counterparts. However, the effect of obesity on exercise‐stimulated muscle protein synthesis remains unknown. Nine lean (23.5 ± 0.6 kg/m^2^) and 8 obese (33.6 ± 1.2 kg/m^2^) physically active young adults participated in a study that determined muscle protein synthesis and intracellular signaling at rest and following an acute bout of resistance exercise. Mixed muscle protein synthesis was determined by combining stable isotope tracer ([^13^C_6_]phenylalanine) infusion with serial biopsies of the *vastus lateralis*. A unilateral leg resistance exercise model was adopted so that resting and postexercise measurements of muscle protein synthesis could be obtained simultaneously. Obesity was associated with higher basal levels of serum insulin (*P *< 0.05), plasma triacylglycerol (*P *< 0.01), plasma cholesterol (*P *< 0.01), and plasma CRP (*P *< 0.01), as well as increased insulin resistance determined by HOMA‐IR (*P *< 0.05). However, resting and postexercise rates of muscle protein synthesis were not significantly different between lean and obese participants (*P *= 0.644). Furthermore, resistance exercise stimulated muscle protein synthesis (~50% increase) in both groups (*P *< 0.001), with no difference between lean and obese (*P *= 0.809). Temporal increases in the phosphorylation of intracellular signaling proteins (AKT/4EBP1/p70S6K) were observed within the exercised leg (*P *< 0.05), with no differences between lean and obese. These findings suggest a normal anabolic response to muscle loading in obese young adults.

## Introduction

Obesity is characterized by well‐described defects in skeletal muscle glucose and lipid metabolism, but few studies have determined the effects of increased fat mass on skeletal muscle protein metabolism. This is an important consideration, as skeletal muscle plays an essential role in metabolic control and healthy aging (Wolfe 2006; McLeod et al. 2016). Muscle mass is determined by the balance between muscle protein synthesis and muscle protein breakdown. Obesity appears to alter these two processes. For example, leg protein turnover (synthesis and breakdown) measured during insulin and amino acid infusion was found to be reduced in obese older men (Murton et al. 2015). Furthermore, the stimulatory effect of protein feeding on myofibrillar muscle protein synthesis was diminished in sedentary overweight and obese individuals when compared with healthy‐weight controls (Beals et al. 2016). Together, these results suggest that an obesity‐induced inability to modulate muscle protein turnover in response to nutrient stimulation might lead to impaired tissue remodeling and reduced quantity or quality of skeletal muscle.

Another potent stimulus for muscle protein turnover is resistance exercise (or mechanical loading) (Phillips et al. 1997), which leads to muscle hypertrophy as a result of cumulative increases in muscle protein synthesis after each bout of exercise (Brook et al. 2015; Morton et al. 2015). Several rodent studies report impaired muscle hypertrophy in obese animals subjected to functional overload (Thomson and Gordon 2006; Sitnick et al. 2009; Paturi et al. 2010). Although interesting from a basic science point of view, we do not believe this to be the case in humans, as obese individuals possess similar, if not greater, lean tissue mass than healthy‐weight controls (Murton et al. 2015; Beals et al. 2016; Smeuninx et al. 2017). Moreover, obese individuals exhibit hypertrophic responses to resistance exercise training (Geisler et al. 2011; Willis et al. 2012). However, to the best of our knowledge, no study has directly compared the anabolic responses (acute or chronic) to resistance exercise conducted in obese versus lean individuals. Thus, the possibility of obesity‐induced anabolic resistance to muscle loading remains to be tested.

Therefore, the primary aim of this experiment was to investigate whether the muscle protein synthesis response to acute resistance exercise was different between lean and obese individuals. To achieve this aim, we combined stable isotope tracer ([^13^C_6_]phenylalanine) infusion with serial muscle biopsies. A unilateral leg resistance exercise model was adopted so that resting and postexercise measurements of muscle protein synthesis could be obtained simultaneously, with both legs exposed to the same systemic milieu. A secondary aim was to assess the phosphorylation status of anabolic signaling proteins (AKT/mTOR/4EBP1/p70S6K) in order to identify possible differences in signal transduction between lean and obese individuals.

## Methods

### Participants

Nine lean (BMI <25 kg/m^2^) and eight obese (BMI >30 kg/m^2^) individuals aged 19–32 years volunteered to participate in this study. In addition to having a calculated BMI >30 kg/m^2^, participants were classified as obese if they had an estimated total body fat ≥25% (male) or ≥35% (female). Participants were nonsmokers, with no diagnosis of cardiovascular or metabolic disease, not taking any medications known to interfere with the study outcomes, and not losing weight (<3 kg weight loss in the past 3 months). Participants were recreationally active but not resistance‐exercise trained. Participants in both groups reported taking part in at least 3 × 30 min of moderate‐intensity physical activity per week. Young, physically active participants were recruited for this study as both aging and inactivity are known to contribute toward anabolic resistance and muscle atrophy. Hence, this study focused on obesity, separate to these other known risk factors. Both males and females were included in this study as the muscle protein synthesis response to exercise (Dreyer et al. 2010; West et al. 2012) and other anabolic stimuli, such as combined hyperinsulinemia and hyperaminoacidemia (Smith et al. 2009), are thought to be similar between males and females. The study was approved by the Loughborough University Ethical Subcommittee for Human Participants (R13‐P90) and was conducted in accordance with the declaration of Helsinki. All participants gave written informed consent after the experimental procedures and possible risks were fully explained.

### Experimental design

Participants attended an initial screening visit to assess their eligibility for inclusion in the study. Upon inclusion in the study, participants attended two further laboratory visits: the first being for assessment of metabolic health, body composition, and leg strength; and the second being a stable isotope tracer ([^13^C_6_]phenylalanine) infusion trial with serial muscle biopsies for assessment of muscle protein synthesis and intracellular signaling responses to an acute bout of unilateral leg resistance exercise. Unilateral leg resistance exercise was chosen for this study so that resting and postexercise measurements of muscle protein synthesis could be obtained simultaneously, with both legs exposed to the same systemic milieu. Additionally, the unilateral approach was chosen over sequential measurements (i.e., resting muscle protein synthesis followed by postexercise muscle protein synthesis) so not to extend the experiment duration unnecessarily, which could have impacted the results by prolonging the fasting period.

### Preliminary assessment

Participants reported to the laboratory in the morning (07.00–08.00 h) after an overnight fast (≥10 h), having refrained from any strenuous physical activity for at least 48 h. A 10‐mL venous blood sample was obtained for assessment of fasting glucose, triacylglycerol (TAG), cholesterol, C‐reactive protein (CRP) and insulin concentrations. Blood samples were divided equally between tubes containing either EDTA, for collection of plasma, or tubes containing a clotting catalyst, for collection of serum (Sarstedt, Leicester, UK). Anthropometric characteristics (height, body mass, body mass index [BMI], and skinfold thickness) were then assessed. Skinfold thickness of the biceps, triceps, subscapular, and suprailiac was measured in duplicate/triplicate to the nearest 0.2 mm using Harpenden skinfold calipers (HaB International Ltd, Warwickshire, UK). Skinfold thickness measurements were then used to estimate total body fat percentage (Durnin and Womersley 1974). Bioelectrical impedance analysis (BIA) was performed as a secondary estimate of total body fat percentage (Bodystat 1500; Bodystat Ltd, Douglas, UK). These measurements were performed in accordance with the manufactures guidelines for pretesting standardization. Thereafter, unilateral leg strength was assessed for each leg using a guided‐motion knee‐extension machine (Selection line; TechnoGym, Cesena, Italy). Participants were verbally instructed on correct lifting technique and cadence, which approximately corresponded to 2 sec concentric muscle action, 0 sec pause and 2 sec eccentric muscle action. Following a standardized warm‐up consisting of five repetitions at 5, 10, and 15 kg, participants performed single repetitions of increasing load until failure. A rest period of 3 min was taken between each successive attempt, and the single highest lift was recorded as the participants’ 1 repetition maximum (1RM). The strongest leg was assumed to be the dominant leg and was selected to perform resistance exercise in the main trial; with the weaker, nondominant leg serving as a resting control.

### Experimental trial

**Figure 1:**
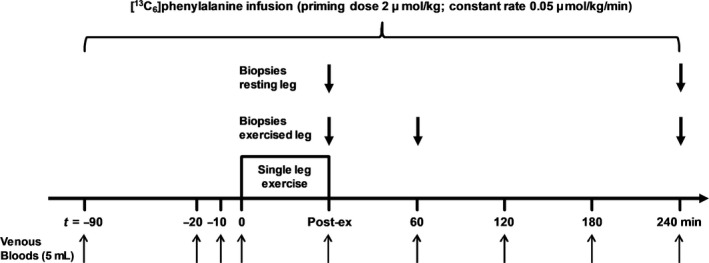
Schematic diagram of the experimental trial and infusion protocol. Arrows indicate the timing of blood and biopsy samples. Exercise was a single‐leg knee‐extension task and consisted of a warm‐up set of 10 repetitions at 30% 1RM followed by four sets of repetitions to failure at 70% 1RM, with 4 min of recovery between each set.

Participants reported to the laboratory in the morning (07.00–08.00 h) after an overnight fast (≥10 h), having refrained from any strenuous physical activity for at least 48 h. After voiding and being weighed, a 20‐gauge Teflon catheter (Becton, Dickinson, Plymouth, UK) was inserted into an antecubital vein of each arm to allow repeated blood sampling and stable isotope tracer infusion. A baseline venous blood sample (5 mL) was obtained for measurement of background isotopic enrichment before a primed constant infusion of L‐[ring‐^13^C_6_]phenylalanine (prime 2 *μ*mol/kg; constant rate 0.05 *μ*mol/kg/min) was initiated and continued for the duration of the experiment (Fig. 1). Further blood samples (5 mL) were obtained 70, 80, and 90 min into the infusion period (referred to as *t* = −20, −10, 0 min in results/figures) in order to confirm isotopic steady state prior to obtaining skeletal muscle biopsies. At this point, 2–3 mL of local anesthesia (Lidocaine 20 mg/mL) was administered to the skin and fascia in a region of the *vastus lateralis* of each leg. A 5–6 mm incision was then made in each leg; this was covered with a sterile dressing that remained in place until completion of the resistance exercise task.

After preparing the two incisions, participants performed unilateral resistance exercise on a guided‐motion knee‐extension machine (Selection line; TechnoGym, Cesena, Italy). Resistance exercise consisted of a warm‐up set of 10 repetitions at 30% 1RM followed by four sets to volitional fatigue at 70% 1RM, with 4 min recovery between sets. The number of repetitions was recorded and volitional fatigue was determined when the participant was unable to perform two consecutive lifts through the full range of motion. Upon completion of resistance exercise, muscle samples (>100 mg) were quickly obtained from the *vastus lateralis* of both the rested and acutely exercised legs using the Bergström needle technique modified for use with suction. Subsequent muscle biopsies were obtained through separate incisions (~3–4 cm proximal to the initial biopsy site) at 60 min (exercised leg only) and 240 min (rested and exercised legs) postexercise. All muscle samples were freed from any visible fat or connective tissue, blotted free of excess blood, and snap‐frozen in liquid nitrogen before being stored at −80°C until analysis. Further blood samples (5 mL) were obtained at 0, 60, 120, 180, and 240 min postexercise.

### Blood analyses

#### Blood metabolites and hormone concentrations

EDTA blood tubes were immediately spun at 1750 *g* in a refrigerated centrifuge (4°C) for 10 min to obtain plasma that was stored at −20°C until analysis. Serum blood tubes were left at room temperature until complete clotting had occurred, with serum separation and subsequent storage being the same as described for plasma. Plasma glucose (#A11A01668), TAG (#A11A01640), cholesterol (#A11A01634), and CRP (#A11A01611) were analyzed using commercially available spectrophotometric assays (all Horiba Medical, Northampton, UK) and a semiautomated analyzer (Pentra 400; Horiba Medical, Northampton, UK). Serum insulin was determined using an ELISA (EIA‐2935, DRG instruments GmBH, Germany).

#### Plasma and intracellular phenylalanine enrichment and amino acid concentrations

Plasma and intracellular ^13^C_6_phenylalanine enrichment was determined alongside quantitative amino acid profiling using liquid chromatography‐tandem mass spectrometry (LC–MS/MS; TSQ Quantiva, Thermo Fisher Scientific, San Jose, CA, USA) as previously described (Borno and van Hall 2014; Borno et al. 2014).

### Muscle tissue analyses

#### Muscle protein enrichment

Mixed muscle protein ^13^C_6_phenylalanine enrichment was determined on 3–5 mg samples of freeze‐dried muscle tissue using gas chromatography‐combustion‐isotope ratio mass spectrometry (GC‐C‐IRMS, Hewlett Packard 5890‐Finnigan GC combustion III‐Finnigan Deltaplus; Finnigan MAT, Bremen, Germany); see previous papers for full details (Hulston et al. 2011; Borno et al. 2014).

#### Intracellular signaling

To investigate intracellular signaling in skeletal muscle, commercially available antibodies were used to determine the phosphorylation of key anabolic signaling proteins (AKT^Ser473^ [Cell Signaling #4060], mTOR^Ser2448^ [Cell Signaling #5536], p70S6K^Thr389^ [Cell Signaling #9234] and 4EBP1^Thr37/46^ [Cell Signaling #2855]; New England BioLabs, Hitchin, Hertfordshire, UK) by SDS‐PAGE and Western blotting.

Muscle samples (30–50 mg wet weight) were homogenized in 10 *μ*L/mg ice‐cooled buffer (1X PBS containing 1% Triton X‐100, 1% protease and phosphatase inhibitor cocktail (Halt protease and phosphatase inhibitor cocktail 100X; Thermo Fisher Scientific, Rockford, IL, USA), and 1% 0.5 mol/L EDTA) using a TissueLyser II and 5 mm stainless steel beads (Qiagen, Hannover, Germany). More specifically, muscle samples were disrupted by the TissueLyser II during 3 × 2 min cycles at 20 Hz. Homogenates were then centrifuged for 10 min at 13,300*g* and the protein content of the resulting supernatant was determined using a Pierce 660 protein assay (Pierce Biotechnology, Rockford, IL, USA). After protein determination, homogenates were mixed with NuPAGE 4X LDS sample buffer (25% final volume; Invitrogen, Carlsbad, CA, USA), *β*‐mercaptoethanol (5% final volume) and distilled water (adjusted for each sample) to prepare final gel‐loading samples with a protein concentration of 1.5 *μ*g/*μ*L. Samples were then boiled at 95°C for 5 min before being stored at −20°C until analysis.

Protein samples (15–20 *μ*g) were loaded onto SDS‐PAGE gels. Depending on the target of interest, proteins were separated using either NuPAGE 10% Bis‐Tris gels or NuPAGE 3–8% Tris‐acetate gels and appropriate running buffers (Invitrogen, Carlsbad, CA, USA). Proteins were then transferred (2 h at 30 V) to polyvinylidene difluoride (PVDF) membranes (Invitrogen, Carlsbad, CA, USA), before blocking for 1 h at room temperature (in Tris‐buffered saline with Tween [TBST] + 5% nonfat dry milk [NFDM] for AKT^Ser473^, p70S6K^Thr389^, and 4EBP1^Thr37/46^ or 5% bovine serum albumin [BSA] for mTOR^Ser2448^). Membranes were then incubated overnight at 4°C with the primary antibody and the appropriate TBST blocking buffer (TBST + 5% NFDM 1:5000 for AKT^Ser473^, TBST + 2% NFDM 1:1000 for p70S6K^Thr389^ and 4EBP1^Thr37/46^, and TBST + 2.5% BSA 1:2000 for mTOR^Ser2448^). Thereafter, membranes were washed four times (3 × 5 min and 1 × 15 min) in TBST before incubation with horseradish peroxidase‐conjugated secondary antibody (Cell Signaling #7074; New England BioLabs, Hitchin, Hertfordshire, UK) for 1 h at room temperature (TBST + 5% NFDM 1:2000 for AKT^Ser473^, TBST + 2% NFDM 1:2000 for p70S6K^Thr389^ and 4EBP1^Thr37/46^ and TBST + 2.5% BSA 1:2000 for mTOR^Ser2448^). Membranes were then washed four times (3 × 5 min and 1 × 15 min) in TBST. SuperSignal West Dura Chemiluminescent substrate (Thermo Fisher Scientific, Rockford, IL, USA) was used as the detection system. Bands were visualized using a Molecular Imager ChemiDoc XRS+ (Bio‐Rad Laboratories, Richmond, CA, USA) and quantified using Quantity One image‐analysis software (version 4.6.8; Bio‐Rad Laboratories, Richmond, CA, USA). Protein phosphorylation was normalized to the total protein loaded per lane using Coomassie staining in order to control for any variation in loading of samples.

### Calculations

The homeostatic model assessment of insulin resistance (HOMA‐IR) was calculated from fasting plasma glucose and fasting serum insulin concentrations (Matthews et al. 1985):

$$\text{HOMA‐IR}=\frac{\text{glucose}\times \text{insulin}}{22.5}$$

where glucose and insulin concentration units are mmol/L and *μ*IU/mL, respectively.

Mixed muscle protein fractional synthesis rates (FSR) were calculated using the standard precursor‐product method:

$$\text{FSR}=\frac{\Delta E_{p}\text{phe}}{E_{\mathrm{ic}}\text{phe}\times t}\times 100$$

where ∆*E*_p_ phe is the change in protein bound phenylalanine enrichment between two biopsies, *E*_ic_ phe is the intracellular phenylalanine enrichment in the second biopsy, and *t* is the period for tracer incorporation in hours. A factor of 100 is used to express FSR in percentage per hour (%/h).

### Statistical analysis

All data are presented as means ± SEM. Statistical analysis was performed using Prism 7 (GraphPad Software, La Jolla, CA, USA). Between group characteristics were compared using an independent samples *t*‐test. Muscle protein FSR and amino acid concentrations and enrichment were compared using two‐way repeated measures ANOVA. Correlational analysis was performed using a two‐tailed Pearson correlation coefficient test. For intracellular signaling, the rest leg and exercise leg were analyzed separately due to unequal time points (i.e., the 60 min time point in exercised leg only). While the ideal statistical analysis for this secondary outcome measurement would have been a three‐way ANOVA, ethical considerations and the lack of any physiological reason for an extra biopsy within the resting leg ultimately determined the study design. Tukey honest significant difference post hoc was applied where appropriate. Statistical significance was accepted where *P *< 0.05.

## Results

### Anthropometric and metabolic health characteristics

Group characteristics are presented in Table 1. Body mass, BMI, sum of four skinfolds, and estimated total body fat percentage were all significantly higher in obese versus lean participants (*P *< 0.001). Furthermore, obesity was associated with elevated plasma TAG, cholesterol, CRP and serum insulin concentrations, and increased HOMA‐IR (*P *< 0.05).

**Table 1:** Anthropometric and metabolic health characteristics of lean and obese participants

	Lean	Obese	Significance (*P*)
Participants (male/female)	8/1	7/1	–
Age (years)	27 ± 1	24 ± 2	0.126
Height (cm)	177.0 ± 3.2	176.3 ± 3.2	0.875
Body mass (kg)	73.8 ± 3.3	105.3 ± 6.9	<0.001
Body mass index (kg/m^2^)	23.5 ± 0.6	33.6 ± 1.2	<0.001
Sum of 4 skinfolds (mm)	31.4 ± 3.2	109.8 ± 11.2	<0.001
Estimated total body fat (%)	13.6 ± 1.9	29.5 ± 1.7	<0.001
HOMA‐IR	2.8 ± 0.3	4.7 ± 0.8	0.021
Fasting plasma glucose (mmol/L)	5.1 ± 0.2	5.5 ± 0.2	0.104
Fasting serum insulin (pmol/L)	85 ± 9	132 ± 22	0.028
Fasting plasma TAG (mmol/L)	0.79 ± 0.08	1.34 ± 0.16	0.003
Fasting plasma cholesterol (mmol/L)	3.84 ± 0.20	4.83 ± 0.23	0.003
Fasting plasma CRP (nmol/L)	3.1 ± 0.6	13.1 ± 3.2	0.004

Data presented are means ± SEM. Measurements of skinfold thickness were performed on the biceps, triceps, subscapular and suprailiac sites. HOMA‐IR, homeostatic model assessment of insulin resistance; TAG, triacylglycerol; CRP, C‐reactive protein.

### Leg strength and performance during the resistance exercise task

Resistance exercise data are presented in Table 2. Single‐leg 1RM for knee‐extension exercise was not significantly different between groups, so there was no difference in the load utilized for the resistance exercise task of the main trial. The number of lifts performed during the resistance exercise task was greater for obese versus lean, but this was not significant. Likewise, there was no difference in the total volume of work performed between groups.

**Table 2:** Leg strength and performance during the resistance exercise task for lean and obese participants

	Lean	Obese	Significance (*P*)
Single‐leg 1RM	60 ± 2	58 ± 4	0.684
70% 1RM	42 ± 2	41 ± 3	0.684
Number of repetitions	37 ± 2	44 ± 4	0.093
Volume of work performed (kg)	1554 ± 111	1792 ± 174	0.129

Data presented as means ± SEM. 1RM, 1 repetition maximum knee extension.

### Plasma and intracellular phenylalanine concentration and enrichment

**Figure 2:**
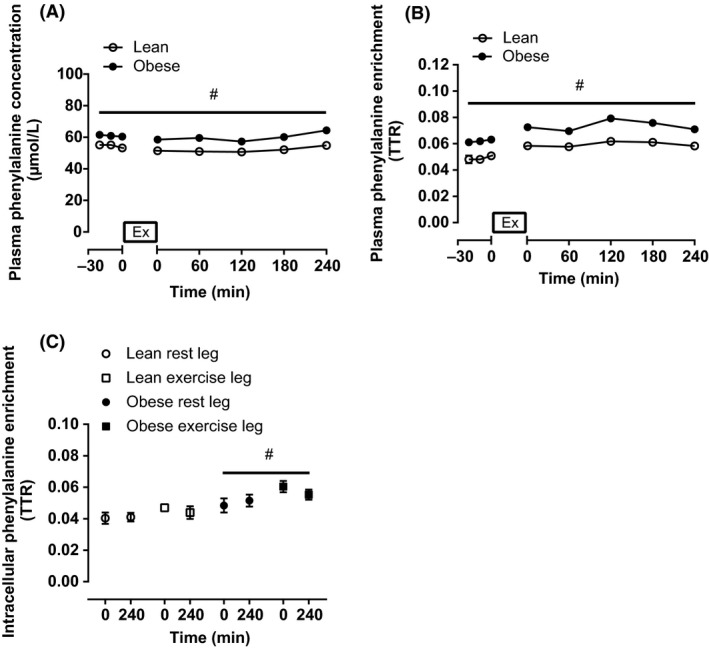
Plasma phenylalanine concentration (A), plasma phenylalanine enrichment (B), and intracellular phenylalanine enrichment (C) during the preinfusion period and following single‐leg knee‐extension exercise for lean (*n* = 9) and obese (*n* = 8) individuals. Values are means ± SEM. Break in the *x*‐axis represents the time period when exercise took place. ^#^Significant difference between lean and obese (*P *< 0.05).

Plasma phenylalanine concentration was stable throughout the experimental trial but was significantly higher in obese than lean (Fig. 2A; *P *= 0.008). The isotope infusion protocol resulted in higher plasma phenylalanine enrichment in obese than lean (Fig. 2B; *P *< 0.001); this effect was also observed in the intracellular environment (Fig. 2C; *P *= 0.005). Higher enrichment levels can be expected as the isotope tracer was administered relative to body mass (not fat free mass [FFM]), and the greater quantity of adipose tissue in obese participants would contribute little toward phenylalanine turnover and isotopic dilution. Intracellular phenylalanine enrichment (i.e., the precursor for FSR calculations) was stable between biopsy time points.

### Mixed muscle protein synthesis and intracellular signaling

**Figure 3:**
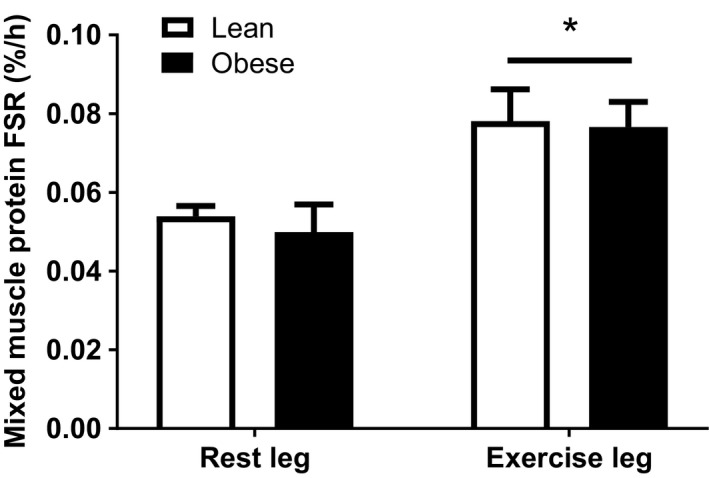
Muscle protein fractional synthesis rates (FSR) in rested and acutely exercised leg muscles of lean (*n* = 9) and obese (*n* = 8) individuals. Values are means ± SEM. *Significantly different to rested leg (*P *< 0.05).

**Figure 4:**
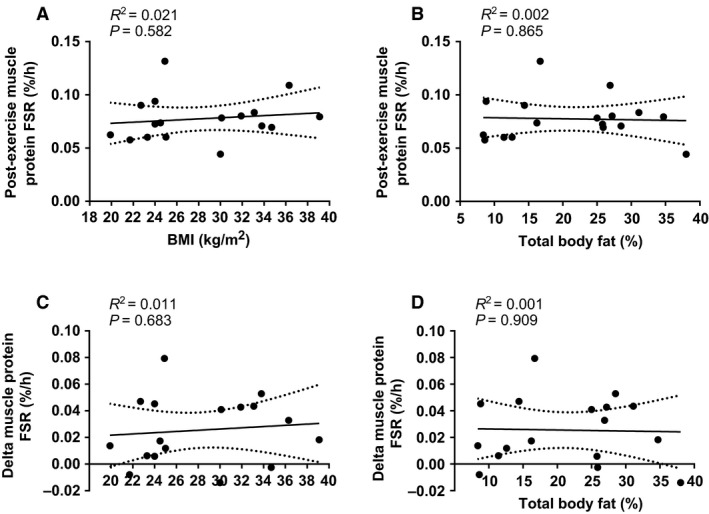
Correlations between BMI (A) and body fat percentage (B) and absolute rates of muscle protein synthesis postexercise, and between BMI (C) and body fat percentage (D) and the change in muscle protein synthesis from rest to post exercise. Data for *n* = 17 in total (9 lean, 8 obese). Dotted lines represent 95% confidence intervals.

Resting and postexercise rates of muscle protein synthesis were not significantly different between lean and obese (Fig. 3; *P *= 0.644). Furthermore, resistance exercise stimulated muscle protein synthesis in both groups (*P *= 0.001), with no difference in the magnitude of this response between lean and obese (*P *= 0.809), suggesting a normal anabolic response to muscle loading in obesity. More specifically, the exercise‐induced increase in muscle protein synthesis was calculated as 45% for lean (from 0.054 ± 0.003%/h [rest leg] to 0.078 ± 0.008%/h [exercised leg]) and 54% for obese (from 0.050 ± 0.007%/h [rest leg] to 0.077 ± 0.006%/h [exercised leg]). An identical response was observed when using the plasma precursor pool to calculate muscle protein synthesis rates (data not shown). Likewise, the muscle protein synthesis responses were unaffected by the inclusion/exclusion of data from the two female participants. As well as assessing group differences in muscle protein synthesis, we performed correlational analysis between BMI (and total body fat percentage) and muscle protein synthesis (Fig. 4A–D). From this analysis, it was clear that no relationship existed between obesity (BMI or body fat percentage) and the muscle protein synthesis response to resistance exercise (either absolute rates postexercise, or the change from rest to postexercise).

**Figure 5:**
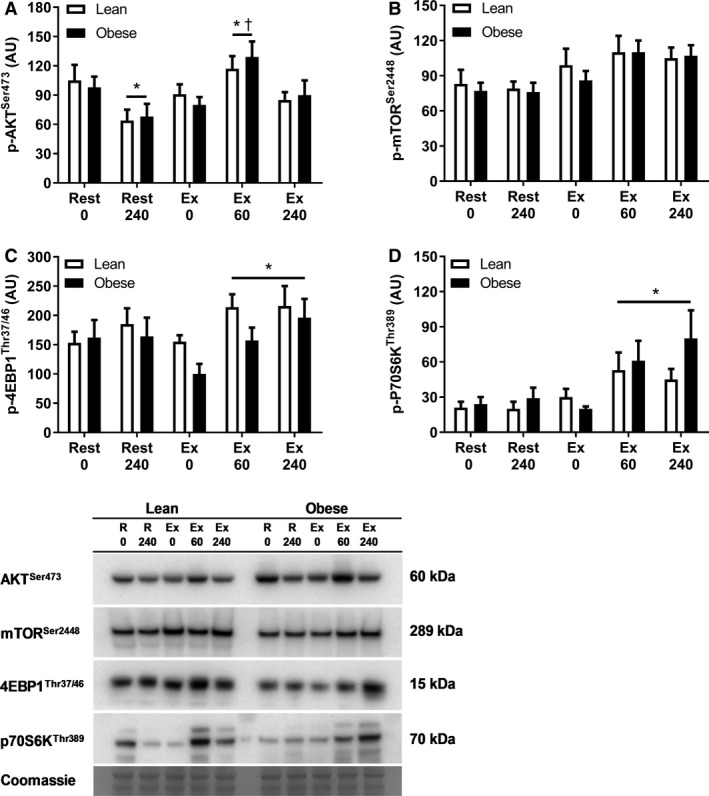
Phosphorylation (p) of AKT^S^ ^er473^ (A), mTOR^S^ ^er2448^ (B), 4EBP1^Thr37/46^ (C) and p70S6K^T^ ^hr389^ in rested (Rest) and exercised (Ex) leg muscles at 0, 60 and 240 min postexercise in lean (*n* = 9) and obese (*n* = 8) individuals. Representative Western Blot images also included. Membranes were cut posttransfer to enable probing for multiple proteins of interest on a single gel. AU, arbitrary units. Values are means ± SEM. *Significantly different to 0 min within same leg (*P *< 0.05). ^†^Significantly different to 240 min within same leg (*P *< 0.05).

Within the rest leg, phosphorylation of mTOR^Ser2448^, 4EBP1^Thr37/46^ and p70S6K^Thr389^ remained stable throughout the experimental trial (Fig. 5B–D), whereas phosphorylation of AKT^Ser473^ decreased from 0 to 240 min (Fig. 5A; *P *< 0.05). Temporal increases in the phosphorylation of AKT^Ser473^, 4EBP1^Thr37/46^, and p70S6K^Thr389^ were observed within the exercised leg (Fig. 5A, C and D; *P *< 0.05), with no differences in these responses between lean and obese (*P *>* *0.05), suggesting that the molecular regulation of loading‐induced stimulation of muscle protein synthesis is not impaired by obesity.

## Discussion

The main finding of this study was that obese young adults responded to an acute bout of resistance exercise with a similar increase in muscle protein synthesis to that of their lean counterparts.

Previous studies have reported a diminished muscle protein synthesis response to nutrient stimulation in overweight/obese individuals when compared with healthy‐weight controls (Murton et al. 2015; Beals et al. 2016). Despite this, excessive fat mass did not appear to negatively influence lean mass in either study or impair muscle function in the study by Murton et al. (2015). In that study, a reduced ability to increase muscle protein synthesis in response to nutrient stimulation was offset by a greater reduction in leg protein breakdown, which may have been a protective mechanism to preserve muscle mass and function. Others, however, have suggested that a decrease in muscle protein turnover might signify impaired tissue remodeling and perhaps contribute to the development of the metabolic dysfunction (Beals et al. 2016). An alternative explanation for why lean mass is often preserved or even greater in obese versus lean individuals is that obese individuals are subjected to greater loading forces and increased contractile work during activities of normal daily living (e.g., standing, walking, stair climbing), which might elicit a physical training effect (James et al. 1978; Murton et al. 2015; Beals et al. 2016; Smeuninx et al. 2017).

Previously it has been shown that overweight/obese individuals are able to mount an anabolic response to several weeks of resistance exercise training, with reports of muscle fiber hypertrophy (Geisler et al. 2011) and an increase in lean tissue mass (Willis et al. 2012). More recently, Stuart et al. (2017) reported that although 16 weeks of resistance exercise training led to an increase in muscle strength and muscle fiber cross‐sectional area within obese, prediabetic men there were no improvements in fasting glucose or insulin concentrations or clamp‐derived measures of insulin sensitivity (so resistance exercise training was only partially effective). Unfortunately, none of these studies included a healthy‐weight control group, so it is not clear if the magnitude of response (i.e., the effectiveness of training) would have been different had the training been undertaken by nonobese individuals. In this regard, we are the first to demonstrate a similar adaptive response, albeit an acute response, to resistance exercises undertaken by obese and lean individuals. This is particularly clear from the correlational analysis presented in Figure 4A–D, which shows that neither BMI (range: 21.7–39.1 kg/m^2^) or total body fat percentage (range: 8.4–38%) had any bearing on the absolute rate of muscle protein synthesis after exercise, or the change from rest to postexercise.

The reason for obesity‐induced impairments in nutrient‐stimulated muscle protein synthesis, but not exercise‐stimulated muscle protein synthesis is not entirely clear, as both stimuli are thought to share a common anabolic signaling pathway (i.e., the ATK/mTOR pathway). However, the molecular regulation of muscle protein synthesis is not yet fully understood. The age and physical activity of our participants is a potential factor for consideration, as obesity‐related anabolic resistance has been reported in older men (55–75 years) (Murton et al. 2015) and sedentary young individuals (Beals et al. 2016) only. This is an important point, as aging (Volpi et al. 2000; Moore et al. 2015) and inactivity (Glover et al. 2008; Drummond et al. 2012; Breen et al. 2013; Wall et al. 2013) are strongly associated with anabolic resistance. So, despite our obese participants displaying some of the classical hallmarks of the metabolic syndrome (i.e., elevated fasting insulin, increased insulin resistance, and dyslipidemia), in addition to increased systemic concentrations of the inflammatory mediator CRP, it is possible that being young and physically active may have mediated some protection against obesity‐related anabolic resistance (to exercise or nutrition). Thus, obesity may not be a problem for muscle protein metabolism unless present under situations of aging, inactivity, or other known risk factors for anabolic resistance.

There are a number of limitations to the present work that require some open discussion. First, we do not have a direct measurement of physical activity for our participants (only the self‐reported physical activity necessary to meet the inclusion criteria). While both groups reported being physically active, it is quite likely that the obese individuals were less active than the lean individuals, but still more active than the older obese individuals recruited in previous studies. Second, it is worth noting that acute measurements of muscle protein synthesis are not always quantitatively predictive of long‐term gains in muscle mass (Mitchell et al. 2015), so in this regard our data should be viewed as preliminary, with future studies being necessary to determine chronic adaptation/the eventual change in phenotype. As a final point for consideration, our experimental trial was conducted under fasted conditions in order to study the isolated effects of exercise (much like previous experiments have studied the isolated effects of nutrition (Beals et al. 2016; Murton et al. 2015; Smeuninx et al. 2017)). Although the provision of protein‐containing foods around the time of exercise (either before or after) may be more reflective of real‐world practice, this approach would have prevented us from drawing firm conclusions regarding the effect of obesity on exercise‐stimulated muscle protein synthesis.

In conclusion, we have shown that obese young adults respond to an acute bout of resistance exercise with an increase in muscle protein synthesis that is comparable to that of their lean counterparts. We also found no evidence for obesity‐induced impairments in anabolic signaling. In future studies, it would be useful to determine whether resistance exercise training translates into equal gains in muscle mass (i.e., chronic adaptation) between lean and obese young and old populations. Additionally, it would be interesting to establish whether resistance exercise could be used to overcome the anabolic resistance to nutrient stimulation previously reported in obesity.

## Conflict of Interest

The authors declare no conflict of interest.
